# Clinical and Epidemiologic Features of Diarrheal Disease due to *Aeromonas hydrophila* and *Plesiomonas shigelloides* Infections Compared with Those due to *Vibrio cholerae* Non-O1 and *Vibrio parahaemolyticus* in Bangladesh

**DOI:** 10.5402/2012/654819

**Published:** 2012-09-24

**Authors:** Erik H. Klontz, Abu S. G. Faruque, Sumon K. Das, Mohammed A. Malek, Zhahirul Islam, Stephen P. Luby, Karl C. Klontz

**Affiliations:** ^1^Carleton College, One North College Street, Northfield, MN 55057, USA; ^2^International Centre for Diarrhoeal Disease Research, Bangladesh (icddr,b), Dhaka, Bangladesh; ^3^Centers for Disease Control and Prevention, Atlanta, GA, USA; ^4^Office of Food Defense, Communications, and Emergency Response, Center for Food Safety and Applied Nutrition, Food and Drug Administration, 5100 Paint Branch Parkway, College Park, MD 20740, USA

## Abstract

Using data from the International Centre for Diarrhoeal Disease Research, Bangladesh (icddr,b) from 1996 to 2001, we compared the clinical features of diarrhea in patients with stool specimens yielding only *A. hydrophila* (189 patients; 1.4% of 13,970 patients screened) or *P. shigelloides* (253 patients) compared to patients with sole *V. cholerae* non-O1 infection (99 patients) or *V. parahaemolyticus* infection (126 patients). Patients exhibited similar frequencies of fever (temperature >37.8°C), stools characterized as watery, and stools containing visible mucus. Dehydration was observed more often among patients with *V. parahaemolyticus* or *V. cholerae* non-O1 infection. Compared to patients infected with *V. parahaemolyticus*, those with *A. hydrophila*, *P. shigelloides*, or *V. cholerae* non-O1 infection were less likely to report visible blood in the stool and, on microscopic examination, less likely to exhibit stool red blood cell and white blood cell counts exceeding 20 cells per high power field. The proportion of patients reporting subjective cure at the time of discharge was significantly smaller for those infected with *V. parahaemolyticus.* These findings suggest that *A. hydrophila* and *P. shigelloides* produce diarrheal disease that is less severe than that resulting from infection with *V. cholerae* non-O1 or *V. parahaemolyticus.*

## 1. Introduction

*Aeromonas hydrophila *and* Plesiomonas shigelloides* are Gram-negative bacilli within the families *Aeromonadaceae *and *Enterobacteriaceae*, respectively. Although the two bacteria occupy separate taxonomic niches, they share attributes in being widely distributed in freshwater, estuarine, and marine environments, and both have been recognized to cause systemic infection in immunocompromised hosts and to be possible causes of diarrheal disease [1, 2]. Water and food serve as vehicles of transmission for both pathogens [1].

At present, aeromonads are not universally accepted as true enteropathogens [1]. While some reports have ascribed [3] or refuted [4] an etiologic role to aeromonads in diarrheal disease, others [5, 6] have postulated that only certain subsets of aeromonads equipped with genes for enterotoxicity are significantly associated with diarrhea. Several factors have contributed to uncertainty regarding the enteropathogenicity of aeromonads: the lack of recognized outbreaks with clonally distinct isolates recovered from patients and an implicated source [1]; infrequent documentation of person-to-person transmission; a paucity of evidence demonstrating experimental pathogenicity in humans [7]; absence of a good animal model to carry out infection studies [6]; in some reports, overlapping prevalence of aeromonads in patients with diarrhea compared to asymptomatic individuals [8]. In contrast, *P. shigelloides* has been identified as the cause of outbreaks of diarrheal disease [9, 10] and is known to play an etiologic role in travelers' diarrhea [11], dysentery [12], and diarrhea in pediatric [13] and adult [14] outpatients.

Relatively few studies have compared the clinical features of diarrheal illness produced by *A*. *hydrophila* and *P. shigelloides* with those of more established enteric pathogens [15, 16]. Such a comparison conducted in a region with a large number of illnesses could offer insight into clinical nuances associated with each agent. Accordingly, we compared the clinical features of diarrheal disease in persons infected solely with *A. hydrophila* or *P. shigelloides* with those in persons infected solely with *Vibrio cholerae *non-O1 or *Vibrio parahaemolyticus*. We chose the latter pathogens for comparison because, like *A. hydrophila* and *P. shigelloides*, both are transmitted by food and water [17], infect a wide age spectrum of persons [18], and were shown to be prevalent in our study population, patients treated at the International Centre for Diarrhoeal Disease Research in Dhaka, Bangladesh (icddr,b).

## 2. Methods

For over four decades, the International Centre for Diarrhoeal Disease Research in Bangladesh has addressed leading health-related problems through patient care, collaborative research, and extension activities. As a component of its surveillance activities, in 1979, the Centre initiated a systematic sampling program wherein every 25th patient presenting to icddr,b with diarrheal disease was studied in detail for the microbiologic etiology of illness. Employing standard microbiologic procedures [19], from 1996 through 2001, diarrheal stools were tested routinely for *A. hydrophila *and *P. shigelloides* in addition to *Salmonella*, *Shigella*, *Vibrio cholera* O1, *V. parahaemolyticus*, *V. cholerae* non-O1, other *Vibrios*, *E. coli* (enterotoxigenic, enteropathogenic, enteroaggregative, enteroinvasive, and Shiga-toxin-producing), *Campylobacter,* rotavirus, *Entamoeba*, *Giardia*, and *Cryptosporidium*.

Aeromonads were identified using standard bacteriological procedures [20]. Stool samples specifically were enriched in bile broth and subcultured onto taurocholate-tellurite-gelatin agar (TTGA). Transparent oxidase-positive presumptive colonies were tested with *Vibrio* polyvalent and *Vibrio* 0139 antisera, and those that tested negative were identified further based on sugar fermentation, amino acid content, and ability to grow in broth of varying salt concentrations. Isolates were identified as *A. hydrophila* based on sugar fermentation results (esculin, salicin, arabinose, mannitol, sucrose, and inositol), amino acid content (arginine, lysine, and ornithine), and ability to grow in broth of varying sodium chloride concentrations (0%, 6.5%, or 8%). Further characterization of isolates was undertaken using vibriostatic compound disc 0129 (150 *μ*g) and the Analytical Profile Index (API), as necessary.

We analyzed microbiologic results for the period 1996 through 2001 along with select responses to a standard questionnaire administered to patients with diarrheal disease diagnosed and treated at icddr,b during the same period. Among other items, the questionnaire solicited information regarding demographics, clinical features of illness, and microscopic evaluation of diarrheal specimens. Pairwise assessments of responses for patients infected with *A. hydrophila* or *P. shigelloides* were compared with those for patients infected with *V. cholera* non-O1 or *V. parahaemolyticus* using a chi-square or Fisher's exact test to assess differences of statistical significance (*P* value <0.05). We hypothesized that if *A. hydrophila* and *P. shigelloides* were enteropathogens, responses for patients with diarrheal disease from whom these two organisms were recovered from stool specimens would be similar to responses from persons infected with *V. cholerae* non-O1 or *V. parahaemolyticus*. For a variable called “outcome” in the questionnaire, patients were asked to specify subjectively whether they felt “cured” prior to discharge or whether the illness was “continuing.” 

Using daily temperature and rainfall data for Dhaka for the entire study period obtained from the Government of People's Republic of Bangladesh, Bangladesh Meteorological Department (Climate Division), we assessed correlations between total monthly rainfall and mean monthly temperature with the number of patients treated at icddr,b from whom a stool culture yielded only one of the following enteric pathogens: *Aeromonas hydrophila*, *Plesiomonas shigelloides*, *Vibrio cholerae* non-O1, or *Vibrio parahaemolyticus*; correlations were calculated using a threshold for statistical significance defined by a *P* value of less than 0.05. 

## 3. Results

 From 1996 through 2001, stool specimens were collected from 13,970 patients with diarrheal disease for assessment of the pathogens listed above. Among these patients, *A. hydrophila* was the sole organism recovered from 189 (1.4%) patients, *P. shigelloides* from 253 (1.8%) patients, *V. cholerae* non-O1 from 99 patients (0.7%), and *V. parahaemolyticus* from 126 (0.9%) patients. The majority of patients were males (Table 1). Patients with *A. hydrophila* infection were younger than those infected by *P. shigelloides*, *V. cholerae* non-O1, or *V. parahaemolyticus* infections. 

Patients with all four types of infections exhibited similar frequencies of fever (temperature >37.8°C), stools characterized as watery, stools containing visible mucus, maximum number of stools per 24 hours, and duration of illness (Table 1). On the other hand, dehydration was observed more often among patients with *V. cholerae* non-O1 or *V. parahaemolyticus* infection, both groups of which received intravenous rehydration more often than did patients from whom *A. hydrophila* or *P. shigelloides* was recovered. In general, patients from whom *V. parahaemolyticus* was recovered experienced the most severe infections as evidenced by the higher frequency of vomiting and production of stools with visible blood, and the higher percentage of stool specimens for which red or white blood cell counts exceeded 20 cells per high power field; in addition, patients infected with *V. parahaemolyticus* were less likely than their counterparts to report a subjective sense of being cured at the time of discharge. 

**Figure 1:**
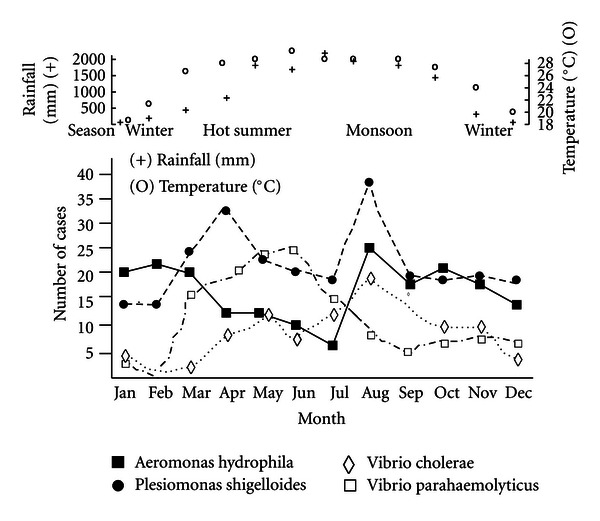
Number of *Aeromonas hydrophila*, *Plesiomonas shigelloides*, *Vibrio cholerae* non-O1, and *Vibrio parahaemolyticus* infections, and mean temperature and total rainfall, by month, International Centre for Diarrhoeal Disease Research, Bangladesh, 1996–2001.

Although pathogens were isolated throughout the year, the monthly number of infections peaked in August for *A. hydrophila*, *P. shigelloides*, and *V. cholerae* non-O1 (Figure 1). The number of infections with *V. cholerae* non-O1 was correlated directly with total monthly rainfall and mean monthly temperature, while the number of *V. parahaemolyticus* infections was correlated with mean monthly temperature (Table 2); no significant correlations were observed with either meteorological variable and *A. hydrophila* or *P. shigelloides*.

## 4. Discussion

In Bangladesh, patients with diarrhea severe enough to require hospitalization were occasionally infected with *A. hydrophila* or* P*. *shigelloides*. Clinically, these patients shared a number of features with those infected with *V. cholerae* non-O1 or *V. parahaemolyticus*. For example, fever (temperature >37.8°C) was rare, mucus was frequently visible in stools, a similar maximum number of stools per 24 hours was reported, and the duration of illness was similar. Moreover, patients infected with *A. hydrophila*, *P. shigelloides*, and *V. cholerae* non-O1 were similar in terms of the frequency with which >20 red blood cells or white blood cells were present per high field on stool examination, as well as in terms of the subjective proportions who deemed themselves to be cured at the time of discharge. 

On the other hand, there appeared to be a gradation of severity such that patients infected with *A. hydrophila* or *P. shigelloides* experienced the least severe illness, while the severity of infection was somewhat greater for *V. cholera* non-O1-infected individuals, and most severe for patients with *V. parahaemolyticus* infection. While a greater proportion of patients with the four infections we studied were males, a finding similar to a report describing *Shigella* infections in Bangladesh [21], those infected with *A. hydrophila* were significantly younger than their counterparts. 

Otherwise, with regard to the visibility of mucus or blood in stools and the presence of >20 red blood cells or white blood cells per high power field, patients infected with *A. hydrophila* and *P. shigelloides* resembled those infected with *V. cholera* non-O1. Moreover, the proportion of patients whose infections were deemed to have been cured was similar for these three groups. However, intravenous hydration as an added component to oral rehydration therapy was used less often among patients with *A. hydrophila* or *P. shigelloides* infections compared to those infected with the *Vibrios*. 

In light of our findings that patients infected with *A. hydrophila* or *P. shigelloides* displayed clinical features that were, in large part, similar to those infected with *V. cholera* non-O1, an organism recognized as a true enteropathogen, we believe there is reason to ascribe a causative role for *A. hydrophila* in diarrheal disease. As others have stated [1, 6], certain strains of *A. hydrophila* likely possess genetic attributes that facilitate the production of a secretory diarrhea in susceptible individuals. Supporting this concept was a study of the distribution of *A. hydrophila* enterotoxins Alt, Ast, and Act in children with and without diarrhea in Bangladesh [22]; the number of isolates positive for both the *alt* and *ast* genes was significantly higher for diarrheal children than for control children, suggesting the enterotoxins produced by the two genes may act synergistically to induce severe diarrhea. Our results corroborate a role for *P. shigelloides* as an etiologic agent of diarrhea as well, an organism that has been associated with outbreaks of diarrhea [9, 10] and, in addition, has been shown to elaborate an enterotoxin that causes elongation of Chinese hamster ovary cells by activating adenylate cyclase with a resulting increase in cyclic AMP, a sequence of physiologic events characteristic of enterotoxins produced by *Vibrio cholerae* and enterotoxigenic *E. coli* [23]. 

We observed several differences in the epidemiology of the four pathogens assessed here. Whereas *A. hydrophila*, *P. shigelloides*, and *V. cholerae* non-O1 infections were diagnosed commonly in infants, *V. parahaemolyticus* infections were distinctly uncommon in this age group, being most often diagnosed in persons aged 15–44 years, a pattern described for *V. parahaemolyticus* cases in Canada as well [24]. The pathogens exhibited divergences in terms of seasonal occurrence too, with no correlation seen between rainfall and temperature with either *A. hydrophila* or *P. shigelloides* infections. In contrast, both *V. cholerae* non-O1 and *V. parahaemolyticus* were correlated with temperature, and *V. cholerae* non-O1 was correlated with rainfall as well. The lack of an observed correlation between *A. hydrophila* and *P. shigelloides* with either rainfall or temperature differs from the results of several other studies. For example, increased recoveries of *Aeromonas* from stool specimens have often been reported during warmer months, a time when concentrations of mesophilic organisms may increase in aquatic environments [1]. In an investigation of a waterborne outbreak of *P. shigelloides* infections in Japan, investigators reported that the recovery of *P. shigelloides* from environmental samples, and mud in particular, was most common during the warmer months [9]. It is possible that absence of a correlation between *A. hydrophila* and *P. shigelloides* infections with either rainfall or temperature in Bangladesh is a result of conditions characteristic of Bangladesh, including climatic, geographic, socioeconomic, sanitary, and host factors. 

A limitation of the present study was that not all known microbial causes of diarrhea were assessed in patients presenting to icddr,b from 1996 to 2001. Thus, it is possible that pathogens other than those screened were, in fact, responsible for a proportion of cases attributed to *A. hydrophila* and *P. shigelloides*. However, if this was the case, the same limitation might apply to patients from whom *V. cholerae* non-O1 or *V. parahaemolyticus* were recovered in pure culture. Arguing against this possibility are clinical and epidemiologic features described here that parallel findings from previous investigations of these two *Vibrios *[15, 24, 25]. Thus, if a substantial number of the illnesses described here were truly sole infections, our paper adds to the evidence of *P. shigelloides* being a cause of diarrhea and strengthens the hypothesis that certain strains of *A. hydrophila* may be true enteropathogens as well. 

## Figures and Tables

**Table 1:** Demographic and clinical features of patients treated at International Centre for Diarrhoeal Disease Research, Bangladesh with stool cultures yielding only *Aeromonas hydrophila*, *Plesiomonas shigelloides*, *Vibrio cholerae* non-O1, or *Vibrio parahaemolyticus*, 1996–2001.

Comparison										
Factor	*A. hydrophila* versus *V. cholerae* non-O1 or *V. parahaemolyticus *					*P. shigelloides* versus *V. cholerae* non-O1 or *V. parahaemolyticus *				
	(*N* = 189)	(*N* = 99)		(*N* = 126)		(*N* = 253)	(*N* = 99)		(*N* = 126)	
	Number (%)	Number (%)	*P* value*	Number (%)	*P* value*	Number (%)	Number (%)	*P* value*	Number (%)	*P* value*
Age: <15 years	111 (59)	43 (43)	<0.02	13 (10)	<0.01	106 (42)	43 (43)	—	13 (10)	<0.01
Male	102 (54)	65 (65)	—	83 (66)	<0.04	150 (59)	65 (65)	—	83 (66)	—
Fever (temp ≥ 37.8°C)	13 (7)	7 (7)	—	6 (5)	—	16 (6)	7 (7)	—	6 (5)	—
Abdominal pain	90 (48)	60 (60)	<0.05	113 (90)	<0.01	149 (59)	60 (60)	—	113 (90)	<0.01
Vomiting	145 (77)	80 (80)	—	118 (94)	<0.01	187 (74)	80 (80)	—	118 (94)	<0.01
Duration diarrhea before arrival: ≤3 days	150 (79)	83 (83)	—	121 (96)	<0.01	209 (83)	83 (83)	—	121 (96)	<0.01
11 + stools/24 hours^±^	92 (49)	38 (38)	—	40 (32)	<0.01	100 (40)	38 (38)	—	40 (32)	—
Dehydration: some/severe	92 (49)	62 (62)	<0.03	110 (87)	<0.01	122 (48)	62 (62)	<0.02	110 (87)	<0.01
Intravenous hydration	44 (23)	38 (38)	<0.01	44 (35)	<0.03	46 (18)	38 (38)	<0.01	44 (35)	<0.01
Watery stool	174 (92)	94 (94)	—	120 (95)	—	223 (88)	94 (94)	—	120 (95)	—
Visible blood in stool	8 (4)	3 (3)	—	37 (29)	<0.01	12 (5)	3 (3)	—	37 (29)	<0.01
Visible mucus in stools	148 (78)	74 (74)	—	110 (87)	—	199 (79)	74 (74)	—	110 (87)	—
>20 red blood cells per high power field (stool)	9 (5)	4 (4)	—	23 (18)	<0.01	16 (7)	4 (4)	—	23 (18)	<0.01
>20 white blood cells per high power field (stool)	43 (23)	24 (24)	—	63 (50)	<0.01	65 (26)	24 (24)	—	63 (50)	<0.01
Duration stay ≤1 day	151 (84)	86 (91)	—	119 (97)	<0.01	219 (90)	86 (91)	—	119 (97)	—
Illness cured	91 (48)	53 (53)	—	43 (34)	<0.02	108 (43)	53 (53)	—	43 (34)	—

*Chi-square test; “—”: *P* value >0.05.

^±^All patients in study had ≥3 stools per 24 hours.

**Table 2:** Correlation coefficients for total monthly rainfall and mean monthly temperature by total number of sole infections per month due to *Aeromonas hydrophila*, *Plesiomonas shigelloides*, *Vibrio cholerae* non-O1, or *Vibrio parahaemolyticus*, International Center for Diarrhoeal Disease Research, Bangladesh, 1996–2001.

Variable	Infection							
	*Aeromonas hydrophila*		*Plesiomonas shigelloides*		*Vibrio cholerae* non-O1		*Vibrio parahaemolyticus*	
	*r*	*P* value	*r*	*P* value	*r*	*P* value	*r*	*P* value
Total monthly rainfall	−0.245	0.44	0.394	0.21	0.858	<0.01	0.397	0.20
Mean monthly temperature	−0.257	0.42	0.542	0.07	0.785	<0.01	0.604	<0.05
